# Measurement of hand grip strength: A cross-sectional study of two dynamometry devices

**DOI:** 10.4102/sajp.v78i1.1768

**Published:** 2022-09-26

**Authors:** Alison Lupton-Smith, Kyla Fourie, Anele Mazinyo, Molebogeng Mokone, Siwelile Nxaba, Brenda Morrow

**Affiliations:** 1 Division of Physiotherapy, Health and Rehabilitation Sciences, Faculty of Medicine and Health Sciences, Stellenbosch University, Cape Town, South Africa; 2 Department of Rehabilitation Sciences, Faculty of Health Sciences, University of Cape Town, Cape Town, South Africa; 3 Department of Paediatrics, Faculty of Health Sciences, University of Cape Town, Cape Town, South Africa

**Keywords:** grip strength, hand grip, hand grip strength, JAMAR® Hydraulic Hand Dynamometer, dynamometry, Camry Digital Handgrip Dynamometer

## Abstract

**Background:**

Grip strength has been identified as an important indicator of health status and predictor of clinical outcomes. The gold standard for measuring grip strength is the JAMAR® Hydraulic Hand Dynamometer. Less expensive dynamometers are available but have not been validated within a hospital setting.

**Objectives:**

To validate the Camry Digital Handgrip Dynamometer (Model EH101) against the validated JAMAR® Dynamometer (Model J00105) in a hospital population.

**Methods:**

A cross-sectional observational study with a randomised single-blind cross-over component was conducted on consenting adult patients admitted to general hospital wards. The best of three measurements taken using the dominant hand was used for analysis.

**Results:**

Fifty-one participants (median [interquartile range] age 42 [30–58] years; *n* = 27 [52.9%] female) were included. The mean difference between the Jamar® and Camry measurements was 1.9 kg ± 3.6 kg (*t*-value 0.9; *p* = 0.4). There was a strong positive correlation between the Jamar® and the Camry devices (*R* = 0.94; *r*² = 0.88; *p* < 0.0001). Excellent agreement was found between Jamar® and Camry measurements (interclass correlational coefficient 0.97, 95% CI 0.94–0.99, *p* < 0.0001). Hand dominance significantly affected the agreement between devices (*p* = 0.002).

**Conclusions:**

The Camry Digital Handgrip Dynamometer is a valid tool for assessing grip strength in hospitalised adult patients.

**Clinical implications:**

The Camry Digital Handgrip Dynamometer could be used as an inexpensive tool to measure grip strength.

## Introduction

Grip strength, which is relatively easy and inexpensive to test, has been shown to be a valid and reliable assessment in healthy people as well as those with disease (Bobos et al. 2020). Grip strength is a significant predictor of important clinical outcomes in a variety of health conditions (Bohannon 2019; Roberts et al. 2011).

Large and multinational studies have reported strong associations between grip strength and all-cause, cardiovascular and noncardiovascular (e.g. respiratory or cancer-related) mortality (Celis-Morales et al. 2018; Leong et al. 2015; Strand et al. 2016). In addition to mortality, hand grip strength has other clinical uses in various population groups. In elderly patients, grip strength can predict fall risk and limitations in activities of daily living (Bohannon 2019; Vermeulen et al. 2011). In various other populations such as those with chronic obstructive pulmonary disease (COPD), postsurgery or hospitalised patients, grip strength can be used as an indicator of nutritional status, length of stay in hospital, exacerbation frequency, hospitalisation, frailty and poor health-related quality of life (Ali et al. 2008; Bohannon 2001; Crook et al. 2017; Cui et al. 2021; Kerr et al. 2006; Leong et al. 2015; Martinez et al. 2017; Norman et al. 2011; Olguín et al. 2017; Pavasini et al. 2019; Puhan et al. 2013). Handgrip strength therefore has the potential to screen for individuals at risk of greater morbidity (Bohannon 2019). The identification of these at-risk individuals may assist in facilitating the streamlining and earlier implementation of suitable rehabilitative interventions.

Grip strength can be tested relatively easily using dynamometry at the bedside (ed. Fess 1992; Roberts et al. 2011). The most frequently used dynamometer in studies, which has been well validated and is considered the gold standard against which other devices are validated, is the JAMAR^®^ Hydraulic Hand Dynamometer (Model J00105, Lafayette Instrument Company, United States of America) (Hogrel 2015; Lee et al. 2020; Mathiowetz 2002). Other dynamometers, such as the Camry Digital Handgrip Dynamometer Model EH101 (Zhongshan Camry Electronic Co., Ltd., China), are less expensive (approx. ZAR100 vs. ZAR5000) and more readily available than the Jamar^®^. They may therefore be more suited for resource-limited environments.

The Camry Digital Handgrip Dynamometer has occasionally been reported in the literature; however, its validity in different populations remains unclear (Wilkinson et al. 2021). A recent study conducted on healthy individuals and community-dwelling elderly individuals in Colombia comparing the Camry Digital Handgrip Dynamometer to the Jamar^®^ found significant concordance and agreement between devices, most notably in those aged 40–49 years (Díaz Muñoz & Calvera Millán 2019). To the best of our knowledge, the Camry Digital Handgrip Dynamometer has not been validated in a clinical setting. If shown to be a valid measurement tool, the Camry Digital Handgrip Dynamometer could be used in clinical practice to aid in identifying patients at risk of adverse outcomes and aid in the prioritisation of the implementation of appropriate rehabilitation to ameliorate the effects of whole-body weakness. Thus, the aim of our study was to determine the correlation and agreement between measures of grip strength using the Jamar^®^ and Camry dynamometers in hospitalised patients in South Africa.

## Method

A cross-sectional observational study, with a randomised cross-over component, was conducted in general wards at Groote Schuur Hospital in Cape Town, South Africa.

Consecutive patients, based on bed numbers, from the randomly selected wards were approached for possible inclusion in our study. Wards were allocated a number and a random number generator (Certified True Randomisers; RANDOM.ORG; Randomness and Integrity Services Ltd., Ireland) was used to randomly select the wards. Patients were considered eligible for participation if they were clinically stable adults who had been in hospital for at least 3 days, able to follow instructions and able to actively achieve a range of 90 degrees of elbow flexion in their dominant arm. Patients were excluded from our study if they had any of the following: acute neck, shoulder, elbow or hand pathology in the dominant arm; chronic conditions such as, but not limited to, arthritis or gout affecting their elbow, wrist or hand; cognitive impairments or the inability to either understand our study information or communicate a decision about participation. Patients were also excluded if they were nonresponsive, receiving inotropic infusions or invasively ventilated.

A sample size of 50 participants was required in order to obtain a power of 80% to reject the null hypothesis of there being poor correlation between the devices at alpha 10%, effect size 0.5 and standard deviation of 1.

### Procedure

Demographic and medical data were obtained from the participants’ medical folders and additional data such as weight, height and hand dominance were measured and captured. Testing order was randomised using a random generator application, in order to reduce bias (Certified True Randomisers; RANDOM.ORG; Randomness and Integrity Services Ltd., Ireland). Single blinding of participants was achieved by ensuring that participants could not see the readings of their results. Furthermore, participants were not told which measurement device was the current gold standard and which device was the cheaper of the two.

Where possible, participants were positioned sitting upright in a chair, with their knees and hips at 90° and with back support. For those unable to mobilise out of bed, the head of the bed was raised as far as possible, ensuring an upright long-sitting position. The shoulder on the dominant side was adducted against the body, the elbow positioned in 90° flexion (unsupported) and the wrist in a neutral position (Balogun, Akomolafe & Amusa 1991; Roberts et al. 2011). Participants were then instructed to grip the dynamometer as strongly as they possibly could, using their dominant hand. Three measurements were taken with each device and the highest value was used in analysis for each device respectively (Roberts et al. 2011). Participants were given a 30-min break before performing the test using identical methods with the other device.

### Statistical analysis

Data were analysed using STATISTICA 13 (StatSoft Inc., Tulsa, USA) and SPSS Statistics version 24 (IBM Corporation, New York, USA). Data were tested for normality using the Shapiro–Wilk test. Frequency tables and descriptive statistics such as means and standard deviations or medians and interquartile ranges (IQR), according to distribution, were used to summarise continuous data. Categorical data are presented as n (%). Pearson’s correlation coefficients (r) were determined for grip strength measures between devices; with *r* > 0.9 defining validity. A Bland–Altman plot was used to determine the level of agreement between the two devices, as well as calculating the intraclass correlation coefficient (ICC). The paired two-sample *t*-test was used to compare grip strength measurements between devices, and a one-way ANOVA was used to determine the effect of multiple variables on grip strength and device agreement. A significance level of *p* < 0.05 was used.

### Ethical considerations

Ethical approval was obtained from the Human Research Ethics Committee of the University of Cape Town (reference number: 175/2018). All included participants provided informed consent.

## Results

Fifty-one participants (median [interquartile range {IQR}] age 42 [30–58] years; *n* = 27 [52.9%] female) were enrolled in our study at a median (IQR) 5 (4–8) days post hospital admission. The median (IQR) BMI was 24.6 (20.8–29.1) kg/m².

Participants’ admission diagnostic category, presence of comorbidities, education level, occupational category and hand dominance are presented in Table 1.

**TABLE 1:** Population characteristics.

Characteristic	*n*	%
**Admission diagnostic category**		
General surgery	22	43.1
Maxillo-facial surgery	10	19.6
Gynaecology	7	13.7
Orthopaedics	5	9.8
Plastic Surgery	3	5.9
Ophthalmology	3	5.9
Urology	1	2.0
Presence of comorbid conditions	23	45.1
**Education level**		
Tertiary education	9	17.7
Grade 12	16	31.4
Grade 8–11	21	41.2
Grade 1–7	4	7.8
**Occupation**		
Unemployed	23	45.1
Pensioner	11	21.6
Sales	5	9.8
Technical	3	5.9
Professional	1	2.0
Clerical	1	2.0
Other	7	13.7
**Hand dominance**		
Right	44	86.3
Left	7	13.7

### Correlation and agreement

The mean strength measurements using the Jamar^®^ and Camry devices were 28.8 kg ± 10.2 kg and 27.0 kg ± 10.1 kg, respectively (*t*-value 0.9; *p* = 0.4). The mean difference between the Jamar^®^ and Camry device scores was 1.9 kg ± 3.6 kg.

**FIGURE 1:**
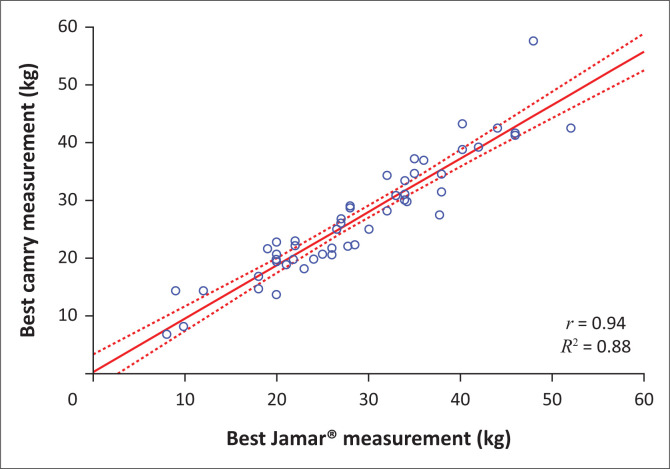
Correlation between measurements obtained using the Jamar^®^ versus the Camry dynamometers (*r* = 0.94; *r*² = 0.88; *p* < 0.0001).

There was a significant strong positive correlation between the measurements obtained using the Jamar^®^ and the Camry devices (*r* = 0.94; *r*² = 0.88; *p* < 0.0001; Figure 1).

**FIGURE 2:**
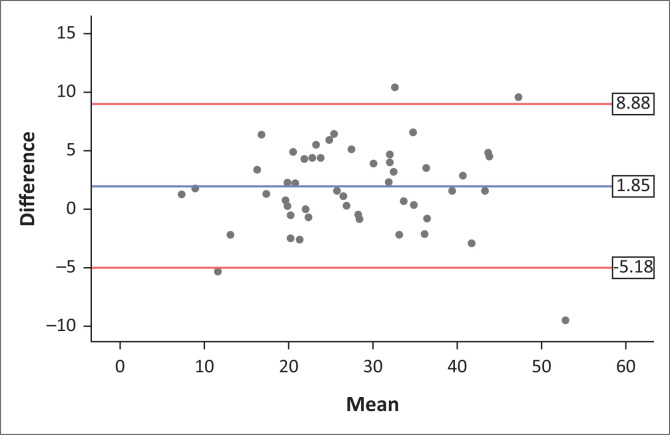
Bland–Altman plot of Jamar versus Camry measurements.

Excellent agreement was shown between the Jamar^®^ and Camry measurements with an intraclass correlation co-efficient of 0.97 (95% CI 0.94–0.98, *p* < 0.0001). The Bland–Altman plot is presented in Figure 2. The mean difference in strength (Jamar^®^–Camry) was small at 1.9 kg, with > 95% of the data points between the acceptable limits of agreement (1.96 × standard deviation [SD] of the mean difference).

### Significance of order of assignment

Twenty-three (45.1%) participants were randomly assigned to using the Jamar^®^ dynamometer first, whilst 28 (54.9%) were assigned to using the Camry device first. Order of assignment had no effect on the difference between Jamar^®^ and Camry measurements (2.3 kg ± 3.2 kg vs. 1.7 kg ± 3.8 kg; *p* = 0.8).

### Effect of gender

Men consistently achieved higher grip strength scores than women, with both devices; however, gender had no effect on the level of agreement between devices, in terms of the mean difference (*F*[1, 47] = 0.17, *p* = 0.68; Table 2).

**TABLE 2:** Handgrip strength according to gender.

Measurement	Male (*n* = 24)	Female (*n* = 27)	*p*
Jamar^®^ (kg)	35.1 ± 9.5	23.3 ± 7.2	< 0.0001
Camry (kg)	32.7 ± 9.7	21.9 ± 7.3	< 0.0001
Jamar–Camry difference (kg)	2.4 ± 4.2	1.4 ± 2.9	0.3

### Effects of diagnosis

Diagnostic category had no significant effect on grip strength, using either device (*F*[1, 47] = 0.073, *p* = 0.79). Similarly, diagnostic category had no effect on the difference between Jamar^®^ and Camry device scores (*p* = 0.60).

### Effect of hand dominance

There was no difference in grip strength between left- and right-hand dominant individuals (Table 3); however, hand dominance had a significant effect on the agreement between the Jamar^®^ and Camry devices (*F*[1, 47] = 7.98, *p* < 0.01).

**TABLE 3:** Handgrip strength according to hand dominance.

Measurement	Right-hand dominant (*n* = 44)	Left-hand dominant (*n* = 7)	*p*
Jamar^®^ (kg)	28.4 ± 9.6	31.9 ± 13.8	0.4
Camry (kg)	27.1 ± 9.9	26.3 ± 11.5	0.9
Jamar^®^–Camry (kg)	1.3 ± 3.3	5.5 ± 3.3	< 0.01

### Effect of age

There was no significant correlation between age and either Jamar^®^ or Camry dynamometry scores (*r* = −0.19 and *r* = −0.20 respectively; *p* = 0.2). Similarly, there was no correlation between age and the difference between the Jamar^®^ and Camry scores (*r* = −0.0003; *p* = 1).

### Effect of body mass index

There was no significant correlation between body mass index (BMI) and either Jamar^®^ or Camry dynamometry scores (*r* = −0.08; *p* = 0.6 and *r* = 0.008; *p* = 0.95). Similarly, there was no significant correlation between BMI and the difference between the Jamar^®^ and Camry scores (*r* = −0.25; *p* = 0.08).

## Discussion

Our results indicate that the Camry dynamometer is a valid tool for measuring grip strength in hospitalised adult patients, with a strong correlation and excellent agreement with the current gold standard, the Jamar^®^ dynamometer.

There was a nonsignificant mean difference in readings between the two devices, with the Jamar^®^ results being marginally higher than the Camry results. This could be attributed to the difference in mechanism and shape of the devices and the feedback that each provides (Amaral, Mancini & Novo 2012; Díaz Muñoz & Calvera Millán 2019). A number of participants reported differences in tactical feedback between the devices. If patients can feel the effects of their gripping efforts displacing the Camry grip piece, then they may stop increasing their grip effort; whereas the lack of tactile feedback from the Jamar^®^ mechanism may result in participants gripping tighter in anticipation of achieving the same ‘give’ or displacement of the hand grip piece (Amaral et al. 2012; Roberts et al. 2011) In studies conducted by Guerra and Amaral (2009) and Amaral et al. (2012), it was noted that discrepancies in results could be attributed to the different ergonomic characteristics and the weight of the respective dynamometers. Similarly, Hamilton, McDonald and Chenier (1992) reported that differing physical make-up of the devices could influence the readings.

Another factor which may account for the difference in readings between the devices is the reading display of the respective devices. The results on the Camry were digital; the grip strength was recorded accurately up to one decimal point, whereas the results on the Jamar^®^ were analogue, in increments of two, leading the authors to estimate in cases where the hand of the device lay between two values. Hamilton et al. (1992) made a similar comment in their study comparing the Jamar^®^ to the sphygmomanometer, stating that their sphygmomanometer had a smaller measurement scale and could therefore detect smaller changes in strength (Hamilton et al. 1992).

An interesting finding was the fact that the differences in measurements between devices was significantly greater in the left-hand dominant participants compared to the right-hand dominant participants. Our study, however, only had a small percentage of left-handed participants, and this finding should be confirmed in larger populations. Although the grip strength achieved by the left-hand dominant and the right-hand dominant participants varied within similar ranges, the average grip strengths of the left-handed participants were slightly higher than the strengths of the right-handed participants (26.3 kg – 31.9 kg versus 27.1 kg – 28.4 kg, respectively), with there being a higher ratio of men to women in the left-hand dominant group. This finding contrasts with various studies which find that on average, right-hand dominant grip strength is higher than left-hand dominant grip strength (Dodds et al. 2014, 2016; Massy-Westropp et al. 2011; Wang et al. 2018). A review conducted by Manoharan, Subramaniam and Jason (2015) found that most of the studies mean values of hand grip strength were higher in right-handed compared to left-handed participants, regardless of gender or posture and joint angle. Left-handed participants in our study were mostly male and younger, which could explain the higher values. Because of the significant differences between the devices in the left-handed participants compared to the right-handed participants, it would be beneficial for future studies to include a larger sample of left-handed people. Additionally, it could be beneficial to assess the agreement between the Camry and the Jamar^®^ in a healthy population.

### Limitations

Our study was directed at hospitalised patients in general wards; therefore, our results cannot be generalised to the general or healthy population of South Africa. Future studies should aim to validate the Camry device across different populations.

## Conclusion

The Camry device has concurrent validity for measuring grip strength in hospitalised patients in South Africa. The Camry can therefore be recommended as an alternative to measure hand grip strength, especially in resource-limited settings. We also found that age, gender and BMI had little to no effect on the measures between the Camry and the Jamar dynamometers; however, hand dominance had an effect. Further studies should be done to assess the agreement between these devices in populations with a larger percentage of left-hand dominant people and in healthy populations.
